# Survival outcomes of First Nations patients with oral cavity squamous cell carcinoma (Poliquin 2014)

**DOI:** 10.1186/s40463-015-0056-8

**Published:** 2015-02-03

**Authors:** Bree Erickson, Vincent L Biron, Han Zhang, Hadi Seikaly, David WJ Côté

**Affiliations:** Department of Surgery, Division of Otolaryngology–Head and Neck Surgery, University of Alberta, 1E4 Walter C Mackenzie Centre 8440-112 Street NW, Edmonton, AB T6G 2B7 Canada; Department of Otolaryngology-Head and Neck Surgery, University of California Davis, Sacramento, California USA

**Keywords:** Head and neck cancer, Oral cavity cancer, First Nations

## Abstract

**Background:**

Oral cavity squamous cell carcinoma (OCSCC) is the most common head and neck cancer, affecting approximately 2000 Canadians yearly. Analysis of Canadian Cancer Registry data has shown that the incidence of oral cavity cancer is decreasing and survival outcomes are improving.

There are significant health disparities in First Nations (FN) people in Canada. The incidence of cancer in FN groups is significantly lower when compared to the general population, but the cancer-related morbidity and mortality is significantly higher. There is no Canadian literature currently for OCSCC, or any other head and neck cancer, that compares survival outcomes of FN to the overall population. Therefore, the objective of this study is to determine whether there is a difference in epidemiology and survival outcomes between FN and non-FN patients with OCSCC.

**Methods:**

This is a retrospective study of a population-based, prospectively-collected database from Alberta Cancer Registry (ACR). Patients with OCSCC, diagnosed and treated in Alberta between 1998 and 2009 were included. ACR data collected included patient gender, age at diagnosis, tobacco and alcohol use, FN status, TNM staging, performance status, date of death, cause of death, and follow-up. FN status was identified through the Alberta Health and Wellness registry and through postal code correlation for those who live on reserves.

**Results:**

A total of 583 patients with OCSCC were included in this study. Of these, 19 were identified as being FN, leaving 564 non-FN patients. When comparing the FN and non-FN groups, there is no significant difference in baseline demographics. Estimated yearly incidences for OCSCC in the Alberta population (all ages) and FN patients are 1.74/100,000 and 1.32/100,000 respectively (p = 0.23). Significant differences are seen in overall survival (OS) (5-year OS 58.1% for non-FN and 33.7% for FN) and for disease-specific survival (DSS) (5-year DSS 67.8% for non-FN and 44.5% for FN). Multivariate analysis confirmed FN patients have a significant increase risk of death in OS and DSS, with hazard ratios of 4.20 (p = 0.01) and 4.57 (p = 0.02), respectively.

**Conclusions:**

The overall survival and disease specific survival are significantly lower in FN patients compared to non-FN patients with OCSCC.

## Introduction

Oral cavity squamous cell carcinoma (OCSCC) is the most common head and neck cancer, affecting approximately 2000 Canadians every year [1]. The most common risk factors associated with OCSCC are tobacco and alcohol use [2]. These factors independently increase the risk of OCSCC, but also have a synergistic effect when used in combination [3]. Overall, the incidence of OCSCC is decreasing, which is thought to be related to decreased smoking rates. Data from the Canadian Cancer Registry between 1992 and 2007 demonstrates a decrease in incidence of oral cavity cancer by 2.1% for men and 0.4% for women [4]. This registry has also shown that survival outcomes are improving for OCSCC with an 8.1% increase in survival for men [4]. These trends of decreasing incidence and increasing survival are mirrored in data from the United States of America [5].

First Nations (FN) people of Canada are defined as those who are associated with a First Nations band and are Aboriginal, without being Metis or Inuit. There are significant health disparities in FN people in Canada based on determinants of health. The incidence of cancer in FN is significantly lower when compared to the general population, but the cancer-related morbidity and mortality is significantly higher. The Alberta Health and Wellness data confirms that between 2000 and 2011, in Alberta, there has been a much higher cancer-related mortality in FN groups. The Canadian literature is very limited, but there is evidence that FN patients do have lower incidence of OCSCC than the overall population. In one study, the Ontario FN patients were reported to have significantly lowered rates of oral cavity cancer from 1968-1991 [6]. There is no Canadian literature currently for OCSCC, or any other head and neck cancer, that compares survival outcomes of FN to the overall population. Therefore, the objective of this study is to determine whether there is a difference in epidemiology and survival outcomes between FN and non-FN patients with OCSCC.

## Methods

Prior to commencement of this study, ethics was obtained from the University of Alberta Health Research Ethics Board (Pro00016426). Inclusion criteria included all patients with OCSCC [7], diagnosed and treated in Alberta between January 1, 1998 to December 31, 2009. Prospectively collected data from the Alberta Cancer Registry (ACR) and clinic charts were used to obtain patient information. The ACR is certified by the North American Association for Central Cancer Registries and is responsible for recording and tracking all new diagnoses of cancer, their treatments and deaths, through mandatory reporting enforced by legislation in the province of Alberta, Canada [8].

Collected information included demographics (age at time of diagnosis, gender, smoking and alcohol status, and FN status), clinical presentation (staging at diagnosis), treatment patterns, functional status (ECOG) [9] and survival outcomes. Smoking status was tabulated from clinic charts and cancer centre charts. Patients with 10 pack year history or more were classified as smokers, given the established prognostic value of this cutoff point [10]. Patients who had quit smoking for 20 years or longer were given a non-smoking status, in accordance with previously published literature [11]. FN status was identified using 2 methods: 1) patients with postal codes associated with one of the 140 Aboriginal Affairs and Northern Development Canada listed reserves and 2) patients with FN status as specified by the Alberta Health and Wellness registry file (registered under the Canadian Federal Indian Act), as described in previous studies [12-14].

### Statistical methods

Survival time was calculated in years from time of pathologic diagnosis to date last known alive by follow-up or electronic medical records, or date of death using a right censoring method. Cause of death was classified as either disease-specific (DSS) or from other causes for calculation overall survival (OS), as determined by the ACR and chart review [15,16].

SPSS version 22.0 was used for all statistical analyses (SPSS Inc., Chicaco, IL, USA). The Kaplan-Meier algorithm was used to estimate overall and disease specific survival, employing the Log-rank test to compare strata [17]. The Cox proportional hazards model [18] was used to perform a multivariate analysis of factors and covariates, including age, gender, TNM staging, tumor subsite, treatment, smoking status and FN status. Pearson’s chi-square and the Mann Whitney *U* test used to calculate differences between groups where appropriate. Standardized incidence ratios (SIRs) were calculated as previously described [19], using census data from Alberta Health and Wellness. Statistical significance was defined as p < 0.05.

#### Ethics approval

Prior to commencement of this study, ethics approval was obtained from the University of Alberta Health Research Ethics Board.

## Results

A total of 583 patients with OCSCC were included in this study. Of these, 19 were identified as being FN, leaving 564 non-FN patients. When comparing patient demographics, none of the baseline parameters were found to be significantly different between the groups (Table 1). The age at diagnosis and gender distribution of non-FN and FN was very similar (61.8 years vs. 60.7 years; p = 0.65 and 56.7% males vs. 36.8% males; p = 0.08, accordingly). The two groups had similar distribution of tumor subsite as well at TNM staging at diagnosis. Rates of tobacco and alcohol use were similar in the groups. Finally, the ECOG scores and treatment modalities employed were not found to be significantly different between FN and non-FN patients.

**Table 1:** **Characteristics of 583 patients with oral cavity squamous cell carcinoma**

**Characteristics**	**All (n = 583)**	**Non-FN (n = 564)**	**FN (n = 19)**	**p** ^**a**^
**Age (mean, SD)**	**61.7**	**61.8**	**60.7**	**0.65**
**Gender (%M)**	**56.1**	**56.7**	**36.8**	**0.08**
**Tumor Subsite (%)**				**0.95**
**Tongue**	**48.0**	**48.0**	**47.3**	
**Gum**	**10.2**	**10.3**	**10.5**	
**Mouth**	**17.3**	**17.4**	**15.7**	
**Floor of Mouth**	**21.9**	**21.8**	**26.3**	
**Palate**	**1.5**	**1.6**	**0**	
**Lips**	**0.9**	**0.9**	**0**	
**TNM Stage (% stage early, I/II)**	**59.5**	**59.5**	**57.9**	**0.88**
**Tobacco (%)**	**67.1**	**66.8**	**76.5**	**0.29**
**Alcohol use (%)**	**64.8**	**64.5**	**80.0**	**0.42**
**ECOG (%)**				**0.73**
**0**	**56.9**	**56.9**	**58.9**	
**1**	**31.6**	**31.4**	**35.2**	
**2**	**7.5**	**7.6**	**5.9**	
**3+**	**4.0**	**4.1**	**0**	
**Treatment** ^**b**^				**0.12**
**S + RT/CRT**	**32.6**	**37.6**	**47.4**	
**CRT**	**1.9**	**2.0**	**0**	
**S**	**52.1**	**52.6**	**36.8**	
**RT**	**7.9**	**7.8**	**10.5**	

SD, standard deviation; %M, percent males; S, surgery; RT, radiation therapy; CRT, chemoradiation therapy.

^a^p-value for Mann–Whitney test comparing FN vs non-FN grouping variables, with significance denoted for values <0.05.

^b^43 patients did not receive treatment (2 FN and 41 non-FN).

The estimated yearly incidence was 1.74/100,000 for all OCSCC patients in Alberta and 1.32/100,000 for FN patients. The mean incidence from 1998–2009 was not significantly different (p = 0.23) between FN and non-FN patients.

**Figure 1:**
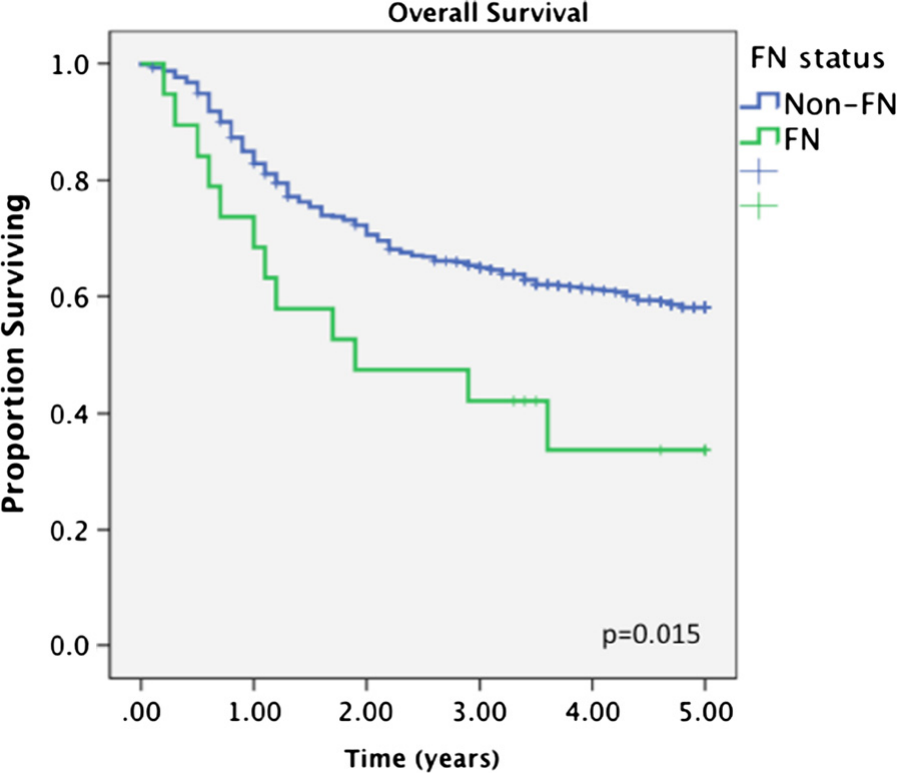
**Title: Overall survival in FN vs non-FN patients.** Legend: At 5 years, First Nations (FN) had an overall survival (OS) of 33.7% compared to 58.1% for non-FN (p < 0.05).

**Figure 2:**
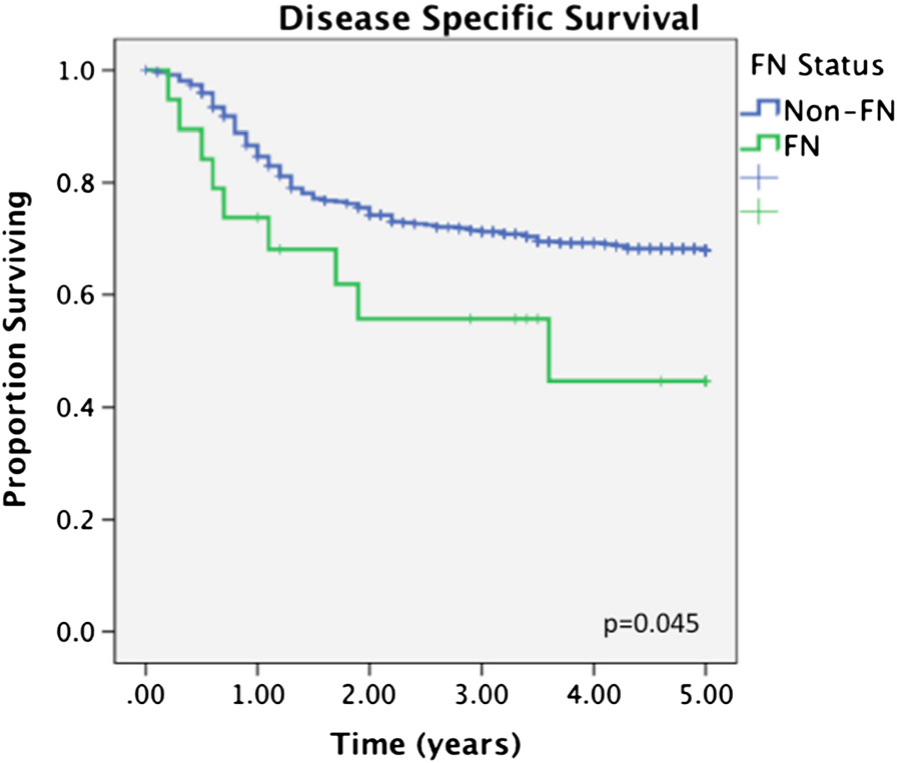
**Title: Disease specific survival in FN vs non-FN patients.** Legend: At 5 years, First Nations (FN) had a disease-specific survival (DSS) of 44.5% compared to 67.8% for non-FN (p < 0.05).

FN patients diagnosed with OCSCC had significantly decreased survival compared to non-FN patients (Figures 1 and 2). At 5 years, FN had an OS of 33.7% compared to 58.1% for non-FN (p < 0.05). Disease specific survival was also decreased for FN patients, with a 5-year rate of 44.5% compared to 67.8% for non-FN (p < 0.05).

Upon multivariable analysis (Table 2), FN status predicted significantly worse survival. Hazards ratio for OS was 4.2 (p = 0.01) and for DSS 4.57 (p = 0.02). The other covariates were not significant predictors of survival.

**Table 2:** **Multivariate analysis**

	**Overall survival**		**Disease specific survival**	
**Covariate**	**Hazard ratio (95% CI)**	**p value**	**Hazard ratio (95% CI)**	**p value**
**Age**	**0.99 (0.97-1.01)**	**0.26**	**0.99 (097–1.01)**	**0.16**
**Female gender**	**0.71 (0.42-1.20)**	**0.20**	**0.61 (0.34-1.11)**	**0.11**
**First Nations (vs Non)**	**4.20 (1.43-12.27**	**0.01**	**4.57 (1.31-15.92)**	**0.02**
**Smoking (vs non-smoker)**	**1.29 (0.65-2.58)**	**0.47**	**1.41 (0.65-3.10)**	**0.39**
**Alcohol use**	**0.65 (0.31-1.36)**	**0.25**	**0.63 (0.27-1.45)**	**0.27**
**Treatment**				
**S + CRT (ref)**	**1**		**1**	
**S + RT**	**0.72 (0.26-1.94)**	**0.51**	**0.76 (0.21-2.71)**	**0.67**
**CRT**	**2.01 (0.39-10.96)**	**0.39**	**1.94 (0.20-19.04)**	**0.57**
**RT**	**4.45 (1.22-16.31)**	**0.02**	**8.43 (1.89-37.63)**	**0.01**
**S**	**1.43 (0.42-4.91)**	**0.57**	**1.42 (0.65-3.11)**	**0.52**
**TNM stage advanced**	**1.34 (0.58-3.10)**	**0.49**	**0.98 (0.39-2.47)**	**0.97**

S, surgery; RT, radiation therapy; CRT, chemoradiation therapy.

## Discussion

The cancer-related mortality in FN people of Canada is significantly higher than in the general population. In this study, FN people with OCSCC in Alberta were found to have significantly worse survival outcomes than non-FN people. The findings are confirmed with both Log-rank testing of 5-year survival rates as well as multivariate analysis. This is the first Canadian study to confirm this relationship, and the results are consistent with published data on indigenous American populations in the United States. Several recent American studies have investigated head and neck cancer in Aboriginal people using the Surveillance, Epidemiology, and End Results (SEER) Program Research Data [20,21]. Dwojak et al., in 2012, found that there was no significant difference in unadjusted survival rates, but that when adjusting for demographic characteristics there was a decreased survival in American Indians/Alaska Natives (AI/AN), with a hazard ratio of 1.3 (p = 0.05).

We did not find any significant difference in incidence of OCSCC between FN and non-FN people of Alberta. This is in disagreement with the sole published Canadian study, which attempted to analyze oral cavity cancer incidence amongst FN people by Marrett and Chaudhry ^6^. The incidence of oral cavity cancer in status Indians in Ontario was found to be significantly lower than non-status Indians with ratio of incidence of 0.98 (95% CI 0.61-1.51) in females and 0.65 (95% CI 0.42-0.97) in males. Further descriptions are found when looking at American data. Reichman et al., in 2008, showed that there was a wide variance of incidence in head and neck cancers amongst AI/AN based on location and specific site of involvement. When examining the oral cavity, it is described that the ratio of AI/AN to non-hispanic white patients was 0.55, 0.76, and 0.70, for tongue, gingiva, and floor of mouth respectively [21].

These discrepancies lead to the concern of FN reporting amongst health data collection as described by Hoopes et al., in 2012 [22]. When there is misrepresentation among relatively small populations, such as FN people, it can significantly affect population-based estimates of incidence and survival. Previous Alberta studies focusing on FN rates of disease have used the Alberta Health Services FN Status Registry alone to identify FN patients [12,14,23]. Our study is the first to use a more inclusive definition by identifying FN patients through FN reserve postal code correlation.

We were able to quantify tobacco and alcohol use amongst patients, although this data is somewhat subjective. Smoking rates in FN people are higher and trending down at a slower rate than amongst the general Canadian population [24,25], but our study did not show any significant difference in smoking rates between the two groups. We believe this is due to very high rates of smoking in both cancer groups when compared to the overall population. We did not include histological grade in our analysis, as historically this has not been proven to be a strong predictor of survival [26,27]. This may be considered a limitation to our study.

The significantly decreased survival outcomes of FN patients with OCSCC in Alberta is concerning and warrants further investigation. A number of possibilities may explain differences between FN and non-FN patients shown here. Access to healthcare, especially in FN patients in remote regions of Alberta, may delay diagnosis and follow-up. Environmental, genetic, and epigenetic factors could be responsible for lower survival outcomes in FN patients but research in this area is currently lacking.

## Conclusion

The incidence of OCSCC is similar for FN and overall populations in Alberta. However, both the OS and DSS are significantly lower in FN compared to non-FN patients with OCSCC. This result has been shown to be independent of variables such as risk factors, stage at presentation, and treatment modalities.

## References

[1] Advisory Committee on Cancer Statistics (2013). Canadian Cancer Statistics 2013.

[2] Hashibe M, Brennan P, Benhamou S, Castellsague X, Chen C, Curado MP (2007). Alcohol drinking in never users of tobacco, cigarette smoking in never drinkers, and the risk of head and neck cancer: pooled analysis in the international head and neck cancer epidemiology consortium. J Natl Cancer Inst.

[3] Blot WJ, McLaughlin JK, Winn DM, Austin DF, Greenberg RS, Preston-Martin S (1988). Smoking and drinking in relation to oral and pharyngeal cancer. Cancer Res.

[4] Johnson-Obaseki S, McDonald JT, Corsten M, Rourke R (2012). Head and neck cancer in Canada: trends 1992 to 2007. J Otolaryngol Head Neck Surg.

[5] Chaturvedi AK, Engels EA, Anderson WF, Gillison ML (2008). Incidence trends for human papillomavirus-related and -unrelated oral squamous cell carcinomas in the United States. J Clin Oncol.

[6] Marrett LD, Chaudhry M (2003). Cancer incidence and mortality in Ontario First Nations, 1968–1991 (Canada). Cancer Causes Control.

[7] Edge SB, Compton CC (2010). The American joint committee on cancer: the 7th edition of the AJCC cancer staging manual and the future of TNM. Ann Surg Oncol.

[8] OConnell D, Seikaly H, Murphy R, Fung C, Cooper T, Knox A (2013). Primary surgery versus chemoradiotherapy for advanced oropharyngeal cancers: a longitudinal population study. J Otolaryngol Head Neck Surg.

[9] Oken MM, Creech RH, Tormey DC, Horton J, Davis TE, McFadden ET (1982). Toxicity and response criteria of the Eastern cooperative oncology group. Am J Clin Oncol.

[10] Ang KK, Harris J, Wheeler R, Weber R, Rosenthal DI, Nguyen-Tân PF (2010). Human papillomavirus and survival of patients with oropharyngeal cancer. N Engl J Med.

[11] Marron M, Boffetta P, Zhang ZF, Zaridze D, Wünsch-Filho V, Winn DM (2010). Cessation of alcohol drinking, tobacco smoking and the reversal of head and neck cancer risk. Int J Epidemiol.

[12] Barnabe C, Joseph L, Belisle P, Labrecque J, Edworthy S, Barr SG (2012). Prevalence of systemic lupus erythematosus and systemic sclerosis in the First Nations population of Alberta, Canada. Arthritis Care Res.

[13] Campbell DJ, Ronksley PE, Hemmelgarn BR, Zhang J, Barnabe C, Tonelli M (2012). Association of enrolment in primary care networks with diabetes care and outcomes among First Nations and low-income Albertans. Open Med.

[14] Deved V, Jette N, Quan H, Tonelli M, Manns B, Soo A (2013). Quality of care for First Nations and non-First Nations people with diabetes. CJASN.

[15] Zhang H, Dziegielewski PT, Biron VL, Szudek J, Al-Qahatani KH, O'Connell DA (2013). Survival outcomes of patients with advanced oral cavity squamous cell carcinoma treated with multimodal therapy: a multi-institutional analysis. J Otolaryngol Head Neck Surg.

[16] Biron VL, O'Connell DA, Seikaly H (2013). The impact of clinical versus pathological staging in oral cavity carcinoma–a multi-institutional analysis of survival. J Otolaryngol Head Neck Surg.

[17] Clark TG, Bradburn MJ, Love SB, Altman DG (2003). Survival analysis part I: basic concepts and first analyses. Br J Cancer.

[18] Bradburn MJ, Clark TG, Love SB, Altman DG (2003). Survival analysis part II: multivariate data analysis–an introduction to concepts and methods. Br J Cancer.

[19] Biron VL, Cote DW, Seikaly H (2011). Oropharyngeal squamous cell carcinoma and human papillomavirus-associated cancers in women: Epidemiologic evaluation of association. J Otolaryngol Head Neck Surg.

[20] Dwojak SM, Emerick KS, Guadagnolo BA, Sargent M, Deschler DG, Petereit DG (2012). Cancer knowledge and screening among American Indians. Otolaryngology-Head Neck Surg.

[21] Reichman ME, Kelly JJ, Kosary CL, Coughlin SS, Jim MA, Lanier AP (2008). Incidence of cancers of the oral cavity and pharynx among American Indians and Alaska Natives, 1999–2004. Cancer.

[22] Hoopes MJ, Petersen P, Vinson E, Lopez K (2012). Regional differences and tribal use of American Indian/Alaska Native cancer data in the pacific northwest. J Cancer Educ.

[23] Barnabe C, Joseph L, Belisle P, Labrecque J, Barr SG, Fritzler MJ (2012). Prevalence of autoimmune inflammatory myopathy in the first nations population of Alberta, Canada. Arthritis Care Res.

[24] Lix LM, Bruce S, Sarkar J, Young TK (2009). Risk factors and chronic conditions among Aboriginal and non-Aboriginal populations. Health Rep.

[25] Ritchie AJ, Reading JL (2004). Tobacco smoking status among Aboriginal youth. Int J Circumpolar Health.

[26] Kearsley JH, Thomas S (1993). Prognostic markers in cancers of the head and neck region. Anticancer Drugs.

[27] Roland NJ, Caslin AW, Nash J, Stell PM (1992). Value of grading squamous cell carcinoma of the head and neck. Head Neck.

